# Modeling Pancreatic Endocrine Cell Adaptation and Diabetes in the Zebrafish

**DOI:** 10.3389/fendo.2017.00009

**Published:** 2017-01-26

**Authors:** Lisette A. Maddison, Wenbiao Chen

**Affiliations:** ^1^Department of Molecular Physiology and Biophysics, Vanderbilt University School of Medicine, Nashville, TN, USA

**Keywords:** zebrafish, glucose homeostasis, β-cell, α-cell, plasticity, regeneration

## Abstract

Glucose homeostasis is an important element of energy balance and is conserved in organisms from fruit fly to mammals. Central to the control of circulating glucose levels in vertebrates are the endocrine cells of the pancreas, particularly the insulin-producing β-cells and the glucagon producing α-cells. A feature of α- and β-cells is their plasticity, an ability to adapt, in function and number as a response to physiological and pathophysiological conditions of increased hormone demand. The molecular mechanisms underlying these adaptive responses that maintain glucose homeostasis are incompletely defined. The zebrafish is an attractive model due to the low cost, high fecundity, and amenability to genetic and compound screens, and mechanisms governing the development of the pancreatic endocrine cells are conserved between zebrafish and mammals. Post development, both β- and α-cells of zebrafish display plasticity as in mammals. Here, we summarize the studies of pancreatic endocrine cell adaptation in zebrafish. We further explore the utility of the zebrafish as a model for diabetes, a relevant topic considering the increase in diabetes in the human population.

## Introduction

Glucose homeostasis is a central physiological mechanism important to maintain proper energy balance and cellular function. While the bulk of the information concerning glucose homeostasis comes from studies in mammals, homeostatic mechanisms are active in many organisms including teleost fish [reviewed in Ref. (1)], which underscores the importance of maintaining tight control of circulating glucose. Maintenance of glucose homeostasis is a coordinated effort between multiple organ systems including the brain, skeletal muscle, liver, and the pancreatic endocrine cells. By appropriately secreting glucagon and insulin into the circulation to regulate the production and uptake of blood glucose, respectively, the pancreatic α- and β-cells play a central role in glucose homeostasis. Interestingly, in conditions where demand for insulin or glucagon exceeds the current secretory capacity, glucose homeostasis is still maintained through increased function and number of α- or β-cells. The regulatory mechanisms of this adaptation or compensation, particularly the compensatory increase of cell number, are not fully defined.

The zebrafish has been firmly established as an attractive animal model to explore questions in developmental biology. This has been aided by the low cost, high fecundity traits of the model (2) as well as the optical transparency (3–6), ease of genome manipulation (7–10), and the amenability toward small molecule screens (11–13). These attractive traits of zebrafish have also inspired investigators to study other biological questions including glucose homeostasis.

## Glucose Homeostasis in Zebrafish

The systems regulating glucose homeostasis in zebrafish are similar to those of mammals in composition, ontogeny, and function. As in mammals, glucose homeostasis in zebrafish involves brain, skeletal muscle, liver, and the pancreatic endocrine cells. For example, the glucose transporter Glut2 has been found to be critical for proper brain development (14) supporting the importance of appropriate glucose control in the brain. In skeletal muscle, glucose transporters are present (15) and glucose uptake has been found to be insulin sensitive (16), suggesting that like in mammals, skeletal muscle is a major site of glucose disposal. The liver has a critical function in glucose homeostasis as it both uses and produces glucose. Gluconeogenesis is dynamically regulated in the zebrafish liver (17), and glucose-regulated *pck* promoter activity has been leveraged to screen for compounds that impact glucose production (18). Furthermore, risk alleles for altered fasting blood glucose in humans have been found to increase gluconeogenesis in the liver (19). This again supports the conservation of regulatory pathways of glucose homeostasis. Coordinating many aspects of glucose homeostasis are the pancreatic endocrine cells. Of primary focus has been the insulin-producing β-cells and the glucagon-producing α-cells. In the zebrafish, these cells are present as early as 1 day post fertilization and their development is regulated by pathways similar to those for mammals (20–22). The conservation of glucose homeostasis system between zebrafish and mammals supports that zebrafish is a relevant model to study mechanisms of glucose homeostasis, including aspects of pancreatic endocrine cell biology.

## Promoting β-Cell Proliferation and Differentiation in Larval Stages with Small Molecules: Lineages and Pathways

Replenishing the β-cell mass has been an active area of investigation for many years as approaches to treat both type 1 and type 2 diabetes. To increase β-cells in adults, often the approach is to manipulate pathways active during development. But there is also a need to understand the mechanisms that promote an increase of β-cells post development, either through neogenesis or through increased proliferation as different mechanisms may be active for adaption to changes in physiology. In embryonic and early larval stages, the pancreatic endocrine cells are primarily coalesced in a single large islet referred to as the principal islet (23). At later larval stages, additional secondary islets are present (24). These secondary islets arise from centroacinar cells in the pancreatic duct (24–28). These cells are Notch sensitive (24, 25, 28) and express markers of endocrine precursors including Nkx6.1 (26) and Nkx2.2 (25). Inducing formation of secondary islets as a way to uncover pathways important in stages beyond early development was the basis of a compound screen (29, 30) and a component of another large-scale screen (30). These screens took advantage of the optical transparency of the zebrafish and transgenic lines that mark the pancreatic endocrine cells. The first screen revealed an important role for retinoic acid signaling in the differentiation of endocrine progenitors (29). Follow-up studies have shown this pathway is functionally conserved in humans (31) and that retinoic acid signaling regulated Sox9b (32), an important transcription factor in endocrine cell differentiation (33). The high-throughput screen was based on increasing endocrine cells in both the principal and secondary islets and yielded several candidate pathways controlling endocrine cell differentiation including NFκB signaling and serotonin signaling (30). Both screens captured changes in both proliferation of endocrine cells and differentiation of precursors. Another compound screen aimed solely to increase β-cell proliferation (34) and relied on expression of markers indicative of the different phases of the cell cycle (35). This screen also identified retinoic acid and serotonin signaling, as well as glucocorticoids, as regulators of proliferation (34). These compound-screening approaches identified both molecules with functions in development, such as Sox9, and also pathways such as serotonin and NFκB which likely also function in post-developmental stages. Ultimately, these compound screening approaches using zebrafish may provide molecules that can be targeted to increase β-cell mass as a treatment for adults with diabetes.

## Pancreatic Endocrine Cell Plasticity in Response to Insufficient Hormone Action

Proper development of the pancreatic endocrine cells is unquestionably crucial to establish homeostatic control. But equally important is understanding the underlying mechanisms that allow adaptation to different physiological stresses, in other words, plasticity. For example, in mammals, the β-cell mass increases during pregnancy (36, 37), with high-fat diet in mice (38–40), and in non-diabetic obese humans (41, 42). With obesity the increase in β-cell mass is an adaptive mechanism to compensate for insulin resistance (43). In type 2 diabetic obese patients, the β-cell mass is decreased compared to non-diabetic counterparts (41, 44), which has been attributed to β-cell death or dedifferentiation, in other words, the loss of β-cell identity (45–47). Zebrafish have been shown to also have β-cell compensatory responses. For example, in states of overnutrition, through culturing in glucose solution or in chicken egg yolk emulsion, the number of β-cells increases (48–53). This treatment also causes β-cell increase in older larvae (52). In juvenile fish, a high calorie diet can promote β-cell proliferation and secondary islet formation (54), indicating that the overnutrition-induced β-cell expansion is not limited only to early larval stages. The compensatory increase in β-cells did not occur with intermittent exposure to the same diets, as would be found in meal-type feeding (52). Consistent with overnutrition as the trigger for the compensatory response, the expansion of the β-cells has been found to be dependent on the nutrient-secretion coupling apparatus in preexisting β-cells (49). Stimulating β-cell secretion through pharmacologic or genetic means increased the number of β-cells in the absence of overnutrition (49). Conversely, reducing β-cell activity inhibited the β-cell expansion in the presence of overnutrition (49). The rapid expansion of β-cells was not through stimulation of β-cell proliferation (49, 52) based on incorporation of EdU, which suggested differentiation of resident precursors. Lineage tracing experiments indicated that these new cells did not arise from the centroacinar cells in the pancreatic duct (50) but arise from cells with *mnx1* and *nkx2.2* promoter activity (50, 52) likely residing within the principal islet. The non-canonically secreted FGF1 has been proposed to be a candidate molecule stimulating differentiation of these resident endocrine precursors (50). Mutation of *fgf1* abolished the overnutrition-induced β-cell expansion but did not alter the baseline β-cell number, and this could be rescued through transgenic expression of human FGF1 (50). Furthermore, when FGF1 was altered to allow for secretion through the canonical secretion pathway, the basal number of β-cells was increased without overnutrition stimulation (50). Intact leptin signaling is important for these responses (53) as leptin receptor mutant larvae had a higher number of β-cells developmentally but did not increase number of β-cells with high-fat diet feeding. In addition, blocking insulin expression through morpholino injection or through expression of a dominant-negative IRS2 protein increased the number of β-cells during embryonic stages (55). Furthermore, in adult fish with skeletal muscle insulin resistance, there was an initial increase in the number of β-cells (16). These studies suggest that with an increased need for insulin function, either due to elevated nutrient intake or through inhibition of insulin signaling, zebrafish increase the number of β-cells as an adaptive mechanism, similar to what has been observed in mammals. These conserved responses indicate that zebrafish are a useful model to study β-cell adaptive mechanisms, and with the utility of zebrafish in genetic and pharmacological approaches, the role of candidate molecules, such as FGF1, can be rapidly assessed.

Although β-cells are often the focus in glucose homeostasis, the glucagon-producing α-cells also have an important role in modulating glucose production. Glucagon acts as a counterregulatory hormone to insulin, and modulating glucagon signaling is becoming an increasingly attractive approach for diabetic treatments (56). It has been found in mice that the number of α-cells increases with blockade of glucagon signaling either by knocking out glucagon, the glucagon receptor, or Gsα, or by impairing glucagon receptor function with antagonists or monoclonal antibody treatment (57–61). With the β-cells, this suggests an adaptive response to the decreased effectiveness of glucagon. This is also true in zebrafish, where mutation of the two glucagon receptors resulted in an increased number of α-cells (62). The adaptive responses to nutritional or hormonal status in both β-cells and α-cells reflect conservation of metabolic responses between mammals and zebrafish. This further indicates that pathways and molecules identified in zebrafish may indeed be relevant to mammals.

## Robust β-Cell Regeneration Following Ablation

Another aspect of the plasticity of pancreatic endocrine cells is regeneration following ablation. Zebrafish has tremendous regenerative capacities, including the β-cells. Similar to compensatory increase of β-cell mass, ablation-induced regeneration is also a response to unmet insulin demand, inferred by the high free glucose levels following ablation (25, 34, 63, 64) and underscores the conservation of β-cell function in zebrafish. Most commonly in zebrafish, β-cells are ablated through β-cell-specific expression of bacterial nitroreductase that converts the prodrug metronidazole to a genotoxic metabolite, resulting in death of the cells (65). There have been other approaches however, including inducible expression of a truncated Bid protein, tBid (49), and mosaic expression of diphtheria toxin (DTA) (66) in larval zebrafish as well as streptozotocin (STZ) treatment in adult fish (67, 68). β-cell regeneration occurs quickly following ablation (25, 65, 69, 70) and has been used as an approach to identify sources of new β-cells. Using lineage tracing in adult fish where β-cells were ablated through nitroreductase/metronidazole, it was determined that the centroacinar cells residing in the pancreatic duct are the primary source of new β-cells based on promoter activity of *nkx6.1* (26) or through Notch responsiveness (25). Ablation of β-cells in larval zebrafish also identified that transdifferentiation of α-cells to β-cells contributes to regeneration (55, 63, 64). Using a combination of pharmacological and morpholino approaches, the α-cell transdifferentiation was found to be dependent on glucagon but not through the modulation of gluconeogenesis (64). This seems to differ from mouse where α- to β-cell transdifferentiation is independent of glucagon signaling (71). The secreted factor IGFBP1 has been found to also enhance α- to β-transdifferentiation following ablation (63). Regeneration following ablation has also been used to identify compounds that increase regeneration (70). This study identified a compound that activates adensosine GPCR to increase proliferation. Interestingly, this compound had a limited capacity to induce β-cell proliferation during development, which may reflect the difference between embryonic immature β-cells and mature β-cells. These studies provide important insights into the origins of and specific pathways leading to new β-cells and exemplify the plasticity of pancreatic endocrine cells. The ablation and recovery studies also exemplify the robust regenerative capacity of zebrafish that is not fully recapitulated in mammalian models. β-cell ablation in mouse using STZ, pancreatic ligation, and partial pancreatectomy causes less robust regeneration (72–77). Understanding the keys that confer the regenerative capacity of zebrafish may provide avenues to boost the regenerative potential in mammals.

## Modeling Diabetes in the Zebrafish

It is always of interest to produce an animal model that accurately reflects a human disease. While studies in zebrafish have been extremely useful to identify molecules, pathways, and cell types that contribute to the plasticity of the pancreatic endocrine cells, to date there have been no models that accurately reflect the life history of a human with diabetes. This is exclusive of models reflecting the maturity-onset forms of diabetes including targeting NeuroD that models MODY6 (78), Pdx1 that models MODY4 (48), and Hnf1ba that models MODY5 (79). However, these forms of diabetes are quite rare in the overall patient population (80). The approaches to mimic type 1 diabetes by ablating β-cells have highlighted the regenerative nature of zebrafish, and hyperglycemia is quickly reversed. Although stable expression of DTA can eliminate all β-cells, these fish have growth retardation and fail to thrive (54). For modeling type 2 diabetes, genetically induced muscle insulin resistance using dominant-negative IGF1 receptor (dnIGFR) expression only resulted in glucose intolerance in aged fish but no elevation in fasting blood glucose (16). Likewise, mutation of insulin receptors specifically in the liver resulted in postprandial alterations in glucose (10) but fasting blood glucose was reduced, similar to the liver insulin receptor knockout mice (81). Overfeeding adult fish quickly results in increased fasting glucose (16, 82) but hyperglycemia was reversed by returning to normal feeding. Although zebrafish are glucose sensitive (16, 83), insulin resistance or overfeeding in and of itself may be insufficient to lead to gross dysfunction of glucose homeostasis. A better understanding of the physiology of glucose control in zebrafish is likely necessary for the development of a truly diabetic zebrafish.

Despite the current lack of a robust model for diabetes, the zebrafish stands to contribute to the understanding of the influence of T2D-associated genetic loci on β-cell mass. Genome-wide association studies have identified loci associated with diabetes risk. The challenge is to determine the relevance of these different loci to phenotypes including β-cell mass and β-cell function and further determine the genes that may be influenced by these loci. Given the genetic tractability, the ease of producing mutations via CRISPR/Cas9, and the proven islet cell plasticity, zebrafish are an extremely attractive model to investigate the role of these candidate loci. Recently, O’Hare et al. examined 67 candidate genes from GWAS studies using morpholino and CRISPR-based approaches (84). The impact on β-cell number and regeneration was assayed, and 25 genes that reduced β-cell number when mutated were found. This included genes previously known to influence β-cell number such as *pdx1* and *pax4* as well as some new genes such as *camk1d*. This study, as well as those using genes underlying monogenic forms of diabetes, supports the utility of zebrafish as a model to study the genetic basis of the disease.

To date, all of the screening modalities have relied on measuring changes in the physical number of the β-cells. While secondary measures have also examined free glucose (30, 34, 84), no primary screen has been done to assay for β-cell function either in parallel or instead of changes in cell number. Approaches to achieve this end are currently lacking. Examining calcium signaling through expression of genetically encoded sensors of calcium activity is one approach that may be useful (85), although difficult to employ in a high-throughput screen. To fully understand the physiology of glucose control in the zebrafish, other assays should be developed, beyond those that rely on cell numbers and free glucose assays.

Given all these measures, studies using zebrafish clearly have contributed to the study of glucose homeostasis. From endocrine cell development, plasticity under different conditions, genetic susceptibility, to modeling diabetes, the zebrafish has and will continue to have utility. With the ever increasing number of patients with diabetes, applying as many resources and approaches can only serve to increase knowledge and provide new avenues for therapies.

## Author Contributions

All authors listed have made substantial, direct, and intellectual contribution to the work and approved it for publication.

## Conflict of Interest Statement

The authors declare that the research was conducted in the absence of any commercial or financial relationships that could be construed as a potential conflict of interest.
